# Community-based health workers implementing universal access to HIV testing and treatment: lessons from South Africa and Zambia—HPTN 071 (PopART)

**DOI:** 10.1093/heapol/czab019

**Published:** 2021-05-08

**Authors:** Lario Viljoen, Tila Mainga, Rozanne Casper, Constance Mubekapi-Musadaidzwa, Dillon T Wademan, Virginia A Bond, Triantafyllos Pliakas, Chiti Bwalya, Anne Stangl, Mwelwa Phiri, Blia Yang, Kwame Shanaube, Peter Bock, Sarah Fidler, Richard Hayes, Helen Ayles, James R Hargreaves, Graeme Hoddinott, J Seeley, D Donnell, S Floyd, N Mandla, J Bwalya, K Sabapathy, S H Eshleman, D Macleod, A Moore, S H Vermund, K Hauck, K Shanaube

**Affiliations:** Department of Paediatrics and Child Health, Faculty of Medicine and Health Sciences, Desmond Tutu TB Centre, Stellenbosch University, Lower Level Clinical Building, Francie van Zijl Drive, Cape Town 7505, South Africa; Department of Sociology and Social Anthropology, Stellenbosch University, Stellenbosch, South Africa; Zambart, School of Public Health, Ridgeway Campus, University of Zambia, Lusaka, Zambia; Department of Paediatrics and Child Health, Faculty of Medicine and Health Sciences, Desmond Tutu TB Centre, Stellenbosch University, Lower Level Clinical Building, Francie van Zijl Drive, Cape Town 7505, South Africa; Department of Paediatrics and Child Health, Faculty of Medicine and Health Sciences, Desmond Tutu TB Centre, Stellenbosch University, Lower Level Clinical Building, Francie van Zijl Drive, Cape Town 7505, South Africa; Department of Paediatrics and Child Health, Faculty of Medicine and Health Sciences, Desmond Tutu TB Centre, Stellenbosch University, Lower Level Clinical Building, Francie van Zijl Drive, Cape Town 7505, South Africa; Zambart, School of Public Health, Ridgeway Campus, University of Zambia, Lusaka, Zambia; Global Health and Development Department, Faculty of Public Health and Policy, London School of Hygiene and Tropical Medicine, London, UK; Department of Public Health, Environments and Society, Faculty of Public Health and Policy, London School of Hygiene and Tropical Medicine, London, UK; Zambart, School of Public Health, Ridgeway Campus, University of Zambia, Lusaka, Zambia; International Center for Research on Women, Washington, DC, USA; Hera Solutions, Baltimore, MD, USA; Zambart, School of Public Health, Ridgeway Campus, University of Zambia, Lusaka, Zambia; Department of Paediatrics and Child Health, Faculty of Medicine and Health Sciences, Desmond Tutu TB Centre, Stellenbosch University, Lower Level Clinical Building, Francie van Zijl Drive, Cape Town 7505, South Africa; Zambart, School of Public Health, Ridgeway Campus, University of Zambia, Lusaka, Zambia; Department of Paediatrics and Child Health, Faculty of Medicine and Health Sciences, Desmond Tutu TB Centre, Stellenbosch University, Lower Level Clinical Building, Francie van Zijl Drive, Cape Town 7505, South Africa; Department of Infectious Disease, Imperial College NIHR BRC, Imperial College London, UK; Department of Infectious Disease Epidemiology, London School of Hygiene and Tropical Medicine, London, UK; Zambart, School of Public Health, Ridgeway Campus, University of Zambia, Lusaka, Zambia; Department of Public Health, Environments and Society, Faculty of Public Health and Policy, London School of Hygiene and Tropical Medicine, London, UK; Department of Public Health, Environments and Society, Faculty of Public Health and Policy, London School of Hygiene and Tropical Medicine, London, UK; Department of Paediatrics and Child Health, Faculty of Medicine and Health Sciences, Desmond Tutu TB Centre, Stellenbosch University, Lower Level Clinical Building, Francie van Zijl Drive, Cape Town 7505, South Africa

**Keywords:** HIV, sub-Saharan Africa, community health workers, universal testing and treatment

## Abstract

The global expansion of HIV testing, prevention and treatment services is necessary to achieve HIV epidemic control and promote individual and population health benefits for people living with HIV (PLHIV) in sub-Saharan Africa. Community-based health workers (CHWs) could play a key role in supporting implementation at scale. In the HPTN 071 (PopART) trial in Zambia and South Africa, a cadre of 737 study-specific CHWs, working closely with government-employed CHW, were deployed to deliver a ‘universal’ door-to-door HIV prevention package, including an annual offer of HIV testing and referral services for all households in 14 study communities. We conducted a process evaluation using qualitative and quantitative data collected during the trial (2013–2018) to document the implementation of the CHW intervention in practice. We focused on the recruitment, retention, training and support of CHWs, as they delivered study-specific services. We then used these descriptions to: (i) analyse the fidelity to design of the delivery of the intervention package, and (ii) suggest key insights for the transferability of the intervention to other settings. The data included baseline quantitative data collected with the study-specific CHWs (2014–2018); and qualitative data from key informant interviews with study management (*n* = 91), observations of CHW training events (*n* = 12) and annual observations of and group discussions (GD) with intervention staff (*n* = 68). We show that it was feasible for newly recruited CHWs to implement the PopART intervention with good fidelity, supporting the interpretation of the trial outcome findings. This was despite some challenges in managing service quality and CHW retention in the early years of the programme. We suggest that by prioritizing the adoption of key elements of the in-home HIV services delivery intervention model—including training, emotional support to workers, monitoring and appropriate remuneration for CHWs—these services could be successfully transferred to new settings.

**KEY MESSAGES**

- When implementing large HIV testing and treatment programmes, community-based health workers (CHWs) will play a key role in supporting implementation at scale.
- With adequate support, newly recruited CHWs implementing an HIV prevention intervention can support good fidelity to community-based testing interventions.
- To ensure optimal effectiveness through a CHW model, implementers should tap into local networks, invest in ongoing training and implement adequate staff supervision and motivation measures.
- To ensure sustainability, health implementers should not only manage performance targets but also monitor, manage and support the emotional capacity of CHWs.

## Introduction

In 2015, the World Health Organization (WHO) recommended that all people living with HIV (PLHIV) be offered HIV testing and antiretroviral therapy (ART) regardless of their CD4 count. This recommendation was operationalized through the ‘universal test and treat’ (UTT) strategy. Given current commitments, 96% of all lower-middle-income countries will have adopted UTT by the end of 2020 (WHO, 2019). Successful implementation holds the potential to reduce HIV incidence and improve patient outcomes (Eaton *et al.*, 2014; Cohen *et al.*, 2016; Kharsany and Karim, 2016). Many sub-Saharan African countries are also moving towards including UTT in their public health policies (Kharsany and Karim, 2016; Ortblad *et al.*, 2019). However, several trials implemented in sub-Saharan Africa have shown that although UTT can lower HIV incidence, on its own, UTT is not enough to reach UNAIDS HIV elimination figures (Baral *et al.*, 2019; Havlir *et al.*, 2020). Even in the context of UTT, challenges included delays in linking PLHIV to care and initiating clients on treatment (Seeley *et al.*, 2019; Havlir *et al.*, 2020). From these findings, it is evident that in addition to the UTT strategy, additional efforts (including community education and active client support) appear necessary if expanded access to HIV testing and treatment and epidemic control is to be achieved in high burden settings (Zeng *et al.*, 2016; Ortblad *et al.*, 2019).

Health specialists have identified the utilization of community-based health workers (CHWs) as one potential strategy to extend HIV-related services. Scott *et al.* (2018, p. 11), in their review of community-based health initiatives, noted that CHWs can ‘help bolster programmes in times of political upheaval, loss of external donor funding and reduced prioritization by the ministry of health’. However, the authors also noted that certain gaps in the literature persist, including on effective strategies to train and supervise CHWs. CHWs have also been included as a key component of the implementation of the interventions evaluated in all four southern African UTT trials (Perriat *et al.*, 2018).

In line with the United Kingdom Medical Research Council (UK MRC) guidance for process evaluation, we used data from the HPTN 071 (PopART) trial to document how implementation of the intervention was achieved in practice, focusing on recruitment, retention, training and support to the CHWs as they delivered services. By analysing quantitative and qualitative study implementation data, we sought to: (i) analyse the fidelity to design of the delivery of the intervention package during the trial, and (ii) suggest key insights for the transferability of the intervention to other settings.

### Community-based health workers in sub-Saharan Africa

In sub-Saharan contexts, CHWs have proven effective for implementing various in-home health services, including the delivery of HIV testing services, linking PLHIV to care and treatment adherence support (Wahl *et al.*, 2019). CHWs have also provided other services, including services related to tuberculosis (Sinha *et al.*, 2019), child care (Schneider *et al.*, 2016; O’Donovan *et al.*, 2018), maternal care (Medhanyie *et al.*, 2012; August *et al.*, 2016), mental health (Magidson *et al.*, 2017), reproductive care (Gullo *et al.*, 2017) and non-communicable diseases (Jeet *et al.*, 2017). CHWs have been especially valuable in contexts where public health facilities face challenges, such as staff shortages (Joseph *et al.*, 2012; Koto and Maharaj, 2016; Schneider *et al.*, 2016; Rao Seshadri and Kothai, 2019). Schneider *et al.* (2008, 2016) noted that, in many contexts, CHWs have managed to hold together otherwise under-resourced and poorly functioning HIV health service systems. In 2008, the WHO identified 313 tasks that are key for effectively addressing the HIV epidemic. Perry *et al.* (2014) argue that 115 of these could be performed by CHWs, including HIV counselling, testing and supporting treatment adherence. Swartz (2013, p. 139); however, notes that, ‘public health policy tends to present CHWs as a homogeneous group, with little attention paid to the nuances of experience, motivation, and understanding, which distinguish these care workers from one another and from other kinds of health workers’.

Extensive CHW programmes have also been implemented in Zambia and South Africa and both countries have a history of active but changing community programmes. Prior to 2010, >23 000 volunteer CHWs assisted with providing health services in Zambian communities. These volunteer CHWs were trained by non-governmental organizations (NGOs) and were available to be absorbed when specific health programmes were implemented. In 2010, the Zambian Ministry of Health (MoH) put forward a proposal to formalize a public sector cadre of CHWs under the National Community Health Worker (NCHW) strategy. This cadre, referred to as Community Health Assistants (CHAs), was created to expand access to health services and were institutionalized through the national health system. Since 2012, the CHAs are regularized, receive formal training and are paid by the Zambian government (Zulu *et al.*, 2015; Shelley *et al.*, 2016; Phiri *et al.*, 2017). The CHA system was intended to provide support for nurses and the CHWs were assigned to spend 4 days a week in communities conducting health screenings and promoting health services; and 1 day a week at the health facilities assisting with task sifting from nurses (Zulu *et al.*, 2015). Services provided by the CHAs include preventive and curative services, reproductive health, child health and support for other medical conditions (Shelley *et al.*, 2016).

In South Africa, since 2011, the CHW programme has been restructured along ward-based primary healthcare outreach teams (WBPHCOTs). The programme is designed to have CHWs deployed in municipal electoral wards in teams, consisting of CHWs supported by nurse team leaders (Schneider *et al.*, 2018). Teams are responsible for health prevention and promotion and to provide support for vulnerable individuals and households. In 2011, there were approximately 72 000 CHWs providing mostly tuberculosis (TB) and HIV services. The purpose of the WBPHCOT programme was to formalize lay CHWs, previously employed through state subsidies by contracted NGOs, ‘to ensure that they are similarly trained, have a clear and standardized scope of work, and become more fully integrated into the district health system’ (Assegaai *et al.*, 2018). Prior to the implementation of the WBPHCOT programme, these workers did not previously work as volunteers and were employed as half-day employees earning stipends, although their status as employees were precarious, and they were poorly managed (Schneider *et al.*, 2018). CHWs often worked with entry-level salaries and their work was seen as a means for gaining general work experience (Nkonki *et al.*, 2011).

## Methods

### Setting

Both Zambia and South Africa have a high HIV burden amongst adults aged 18- to 49-years-old, with HIV prevalence of 11.5% (10.9–12.1%) in Zambia and 19% (16.1–20.9%) in South Africa (UNAIDS, 2019). In this context, the HPTN 071 (PopART) cluster randomized controlled trial was conducted in 21 communities from 2013 to 2018 (Hayes *et al.*, 2019). The aim of the trial was to measure whether the implementation of an HIV prevention package including UTT, delivered at household level, resulted in reduced HIV incidence over time, compared to communities receiving standard of care. A core component of the trial was the ‘universal’ delivery of household HIV testing, counselling and referral services by a newly recruited cadre of CHWs (Hayes *et al.*, 2014). The household testing services were implemented in three annual rounds over the course of the trial, from 2013 to 2018. The trial recruited 737 CHWs, to implement the trial intervention package in the 14 peri-urban intervention study communities. The communities are mostly urban and peri-urban, densely populated and fall within the lower socio-economic bracket. Trial communities were defined as the ‘catchment area of a given public health facility’, and accordingly, health facilities were assumed to be fairly easily accessible to most households in the trial. The CHWs, working in pairs, went door-to-door, offering HIV prevention services and capturing intervention data on electronic data-capturing devices. Intervention implementation was successfully achieved in both countries, and the final trial analysis showed that HIV incidence was 20% lower in communities with intervention-specific CHWs than those without (Hayes *et al.*, 2019).

In both Zambia and South Africa, CHWs have played a central role in supporting health programmes (Twumasi and Freund, 1985; van Ginneken *et al.*, 2010). Historically, CHWs were the frontline of palliative care, grief counselling, and HIV prevention education in the pre-ART era (Schneider *et al.*, 2008). More recently, services led by CHWs have been developed to provide a more comprehensive and long-term range of care (Mwai *et al.*, 2013; Perry *et al.*, 2014; De Neve *et al.*, 2017).

As noted above, CHWs were active in Zambia and South Africa prior to the implementation of the PopART trial and routine CHW services continued in both countries as the trial-employed CHWs provided additional HIV-specific services. However, and as described above, there are notable differences in the structure and management of pre-existing CHWs in the two countries. These differences meant that there was variability in how staff were recruited, managed and retained, to accommodate local institutional requirements, in-country labour laws, existing CHW structures and community-specific needs.

### Data collection processes

We pooled several quantitative and qualitative datasets collected over the course of the trial. The quantitative data were collected as part of the stigma ancillary open cohort study (HPTN 071a) where health workers, including the CHWs employed by the trial, voluntarily participated in three rounds of self-administered electronic surveys from 2014 to 2018 (Hargreaves *et al.*, 2016). We include baseline survey data and data from the enumeration database^1^ where we were able to collect demographic data for all health workers. In the surveys, we collected information on socio-demographic background, training and qualification profiles. Response rates for the survey were 85.6% (631/737) in Round 1, 91.5% (605/661) in Round 2 and 90.5% (636/703) in Round 3.

The qualitative data were collected as part of systematically documenting the ‘story of the trial’ in Zambia and South Africa. We included semi-structured key informant interviews (KII) with study management staff from Zambia (*n* = 55) and South Africa (*n* = 36), conducted over the course of the trial; structured observations of PopART implementation processes, including formal study-related training events for the CHWs (*n* = ∼12); and annual on-the-ground observations of the implementation of the intervention (*n* = ∼68), which included spending time with the intervention teams as they conducted in-home visits.

### Data analysis

We describe the sociodemographic and other salient characteristics of the CHWs by country from the available quantitative data in the health worker baseline and enumeration datasets. Qualitative data, including interview transcriptions and structured observation data, were organized in ATLAS.ti. The thematic analysis (Braun and Clarke, 2014), led by two co-authors in Zambia and two in South Africa, involved iterative steps including reading through transcripts and field notes; identifying key themes; refining themes between co-authors; and follow-up discussions with data collection teams where clarification was needed. In our analysis, we emphasized the differences and similarities between countries.

### Ethical considerations

The study was approved by the London School of Hygiene and Tropical Medicine, University of Zambia, and Stellenbosch University research ethics committees. All participants signed written informed consent in accordance with guidance from the in-country research ethics committee. Interview quotes are ascribed using pseudonyms to protect participant confidentiality.

## Findings

### HPTN 071 (PopART) CHW profiles in Zambia and South Africa

The study initially recruited and trained a total of 737 CHWs across the 14 study communities. The number of staff per study community ranged from 28 to 115, proportional to the study population. Of the 737 CHWs, 631 (405 in Zambia and 226 in SA) participated in Round 1 of the survey (Table 1). Generally, the staff employed in Zambia were older, more likely to be married and were significantly more experienced in providing HIV-related services than those in South Africa. More CHWs were self-reported to be living with HIV in Zambia (105/405, 25.9%) than in South Africa (16/226, 7.1%). In Zambia, the staffing component was reflective of CHWs active in communities outside of the trial (Ministry of Health, 2010). In contrast to trial employees in South Africa, CHWs outside of the trial are mostly older females with some form of previous training (van de Ruit, 2019). In addition to the age difference between trial CHWs and CHWs employed by other programmes in South Africa, trial CHWs had a higher level of education (high-school completion), which was not a requirement for NGO-employed CHWs, prior to the implementation of the WBPHCOT programme (Schneider *et al.*, 2018).

**Table 1:** Demographic and human resources characteristics by country

	Zambia	South Africa
CHWs enumerated		
*N* (Round 1)	443	294
Sex		
Female	281 (63.4%)	232 (78.9%)
Male	161 (36.4%)	62 (21.1%)
Unknown	1 (0.2%)	
Staff retention: Start of Round 1 to Round 2	423/443 (95.5%)	183/294 (62.2%)
Staff retention: Start of Round 2 to Round 3	436/457 (95.4%)	149/203 (73.4%)
CHWs surveyed		
*N* (Round 1)	405	226
Age (median)	38	29
Sex		
Female	256 (63.2%)	183 (81.0%)
Male	149 (36.8%)	43 (19.0%)
Marital status		
Married	232 (57.3%)	58 (25.7%)
Not married	173 (42.7%)	168 (74.3%)
Completed secondary schooling	394 (97.3%)	226 (100%)
Residential address inside community	265 (65.4%)	211 (93.4%)
Experience providing HIV service (average years)	6.6	1

In South Africa, there was considerable staff turnover of CHWs, especially between hiring and the commencement of the second round of the trial intervention. Of the 294 CHWs originally employed in 2014, 111 (37.8%) were no longer working for the study by 2015. In addition, there was a turnover of 26.6% (54/203) from the second and third rounds of intervention in South Africa. In contrast, only 4.5% (20/443) of the enumerated study employed CHWs left between the start of the first and second rounds of the intervention in Zambia, and staff turnover remained the same between the second and the third intervention round (21/457, 4.6%).

### Job requirements and staff recruitment

In both Zambia and South Africa, the study teams were tasked with hiring a large number of new staff members to implement the large-scale intervention. In Zambia, the requirements for employment included experience working in the field of HIV and the completion of some secondary schooling. Because of formal regulations of the academic institution implementing the intervention in South Africa, applicants were required to have completed secondary schooling while previous work experience was not prioritized. As such, applicants in South Africa generally had less work experience than their Zambian counterparts. Different recruitment strategies were followed in the two countries. In Zambia, an independent, external consulting company was appointed to recruit, shortlist, and interview CHW candidates. The process was documented and thoroughly recorded in order to demonstrate transparency to study communities. Consultants were assisted by the local Community Advisory Boards (CABs), institutional intervention managers, Neighbourhood Health Committees (NHCs) and clinic staff. Other researchers have noted the importance of community involvement in the appointment in CHWs and local health programmes (Shelley *et al.*, 2016). However, in South Africa, recruitment, shortlisting and hiring was done through the academic research institution’s routine structures, which potentially impacted the ways in which the cadre of CHWs were received. In both countries, advertisements were placed at the local clinics, libraries, churches, markets and in local newspapers. Because of the involvement of local structures (CABs, NHCs, etc.), the Zambian team were able to effectively recruit from existing volunteer CHWs in a way the South African team were not. At the time of the trial, unemployment rates in South Africa were exceptionally high (25.2% vs 10.1% in Zambia in 2015) (The World Bank, 2020), applicants were often desperate for work and reported that they were ill-prepared for the tasks ahead. For example, upon hearing about the work opportunity, one CHW recalled her reaction to the advert: ‘The only requirements were completed secondary education and if you could work with people’. She added, ‘but we didn’t know exactly what we were going to do in the community’ (GD, SA19, 2014).^2^ Many of those employed had no previous experience of health-related work:

Sometimes we find that our knowledge is limited. Sometimes we meet people [community members] that have more knowledge than we actually have. While we are telling them this, they will be telling us more. You feel like a small person, as if you don’t know what you have come for (GD, SA16, 2016).

The Zambian CHWs, predominantly drawn from a pool of experienced workers listed with the MoH, were more confident in their new positions. When asked about their expectations of their jobs, they noted that they were familiar with the roles and drew on previous experience: ‘It is not all that different since we are psychosocial counsellors. So, the same counselling that we [did before], they [PopART] just added on top of that’ (GD, Z2, 9 September 2015). Even CHWs who anticipated some of the potential challenges associated with community-based work felt comfortable at the start of intervention delivery:

I thought maybe it will be tough and difficult. Community [work] is not an easy thing. Then I find that, ah, at the moment it is very easy. People, they accepted us, we make the rapport and the friendship, and people, they welcome us (GD, Z2, 2015).

In Zambia, the clinic staff and CAB members also actively encouraged PLHIV with experience working in healthcare to apply for positions. As a result, almost a quarter of the CHWs in Zambia self-reported as living with HIV. In South Africa, there was no special recruitment for CHWs living with HIV although, early on in the intervention, South African study staff reported that many community members were under the impression that all CHWs were living with HIV (Field notes from: SA14; SA16; SA18; 2014).

### Training

CHWs received training on the HPTN 071 (PopART) study design; information on sexually transmitted infections; TB; prevention of mother to child transmission; voluntary medical male circumcision (VMMC); adherence support; introductory training on stigma; and, their expected roles in the study. The same training consultants were used in Zambia and South Africa. In Zambia, training was conducted with smaller groups of CHWs while, in South Africa, centralized training was conducted with all CHWs. The trainers found the larger group setting challenging as they felt that the lack of intimacy might have compromised the quality of the training and the opportunity to engage with the CHWs on an individual basis.

In both countries, facilitators noted that there were challenges in translating the scientific rationale of the study into understandable terms. In Zambia, several of the CHWs had previously been trained in couples HIV counselling, children’s HIV counselling, and VMMC training (Zambian Training report, 2013). However, few CHWs were familiar with the concepts of universal access to HIV testing and treatment (UTT) and HIV treatment as prevention (TasP), prior to training. For many CHWs in South Africa, this was their first formal encounter with HIV training. The concept of VMMC, in particular, proved challenging in South Africa. Traditional circumcision has cultural significance to Xhosa people, and the topic was considered taboo for women to speak about and therefore implement in practice.^3^ For example, the CHWs expressed concerns about how community members would react if they (as women) approached men to discuss circumcision:

When we were being employed, we didn’t anticipate, and we didn’t know that we were going to do such a job. Obviously, when we were told, we had our insecurities [wondering], how people will react (GD, SA14, 2014).

All CHWs were given regular (monthly) refresher training sessions focused on addressing ‘frontline’ challenges (e.g. client communication, referrals, data devices, data integrity—see Floyd *et al.*, 2018). Between each of the three annual rounds of intervention delivery, staff received 4–6 weeks of further training. These training sessions focused on follow-up support for households. Additional *ad hoc* training was provided when site-specific issues arose. South Africa teams also received professional safety training to mitigate the challenges of high crime rates after staff raised concerns.

### CHW staff retention

The retention of study employed CHWs was seen as key for the success of the intervention. Over time, their increasing familiarity with the study communities, building trusting relationships with community members, and the regular client support they provided meant that the CHWs were vital to ensuring consistent delivery of HIV services. In addition, the investment in their training and the timeframe of the trial meant that retaining study-employed CHWs was a priority for intervention managers. The reasons for staff turnover were often attributed to non-competitive salaries, lack of job security and general working conditions (e.g. adverse weather conditions; walking long distances)—similar to what has been reported elsewhere (Nkonki *et al.*, 2011).

Salaries were often a point of contention in both countries. Although salaries were later adapted, the South African research institute was initially required to offer salaries for study-employed CHWs that were comparable to those received by the CHWs employed by local NGOs. This was to ensure that the study would not attract CHWs away from organizations already working with staffing constraints. As such, the salaries were relatively non-competitive for recent secondary school-leavers. During a discussion, a key informant noted:

We get resignations every week … They [CHWs] just feel the salary is [so little] and then once they get something [another job] that is [USD 10 more], then they take it and then they give 24-hour notice. It is a big problem (KII, South Africa, 2014)

The South African CHWs confirmed this during group discussions: ‘When you see other jobs with better pay, you just want to apply’ (GD, SA19, 2014). The salaries were both a concern and a source of embarrassment for some of the CHWs in South Africa. One woman noted that:

Most people just want to know how much we [earn], because they say the work that we do … is almost like doctors and they want to know how much [we] get paid because [we] look professional. But we can’t [tell them] … You are shy to get this little pay, you don’t want to say [how little it is] (GD, SA20, 2014).

However, this changed in 2015 with the changes in South African labour laws, which stipulated a national minimum wage for all contracted staff—including all CHWs. This resulted in salaries increasing significantly and fewer resignations in South Africa.

Perceived low salary levels were also mentioned in Zambia. During training events, CHWs expressed dissatisfaction with the initial salary levels. During one interactive feedback session, CHWs were asked to write down their concerns. Several CHWs noted, ‘The money is too little, and the job is so big!’ (Training observations, 2014). In Zambia, CHW initial monthly salaries increased significantly between 2014 and 2017 to account for inflation and an additional ‘cost of living’ allowance provided. In Zambia, salaries included medical insurance, access to educational loans and a ‘gratuity fee’ at the end of their contracts.

Job seeking in South Africa was generally dynamic, with staff looking for more permanent work. Their employment in the study was time-limited, which led to insecurities: as one CHW noted: ‘If the three years are over, what’s going to happen to us?’ (GD, SA20, 2014).

CHWs were also concerned about dangerous and challenging working conditions, particularly in South Africa. One study manager noted:

They know how dangerous the [study communities] are, even though you tell them in the interview it is dangerous and everything … It is one thing to say it and another to experience it on a daily basis (KII, South Africa, 2014).

Staff retention was further complicated by the burden of providing regular care to clients in hazardous conditions while earning low wages. One CHW, reflecting on the emotional burden of care noted:

When [a client] dies, you feel like it is a very close relative that has died, and it pains. We even attended two funerals and it is not something that you expect in this line of work … When someone dies, we really get touched (CHW discussion, Zambia, 2015).

CHW expressed that they were not compensated adequately, nor did they receive the necessary support for either the physical or psychological burden of providing services under these conditions.

### Staff management and support

**Figure 1:**
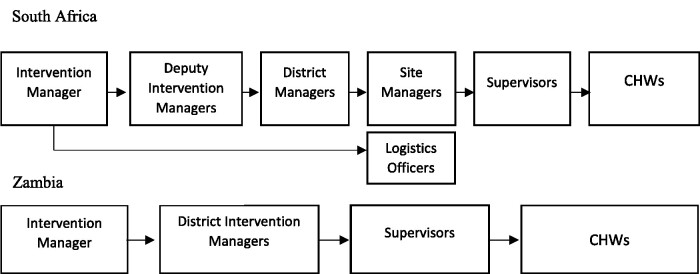
CHW management structure in South Africa and Zambia, after changes were made.

Both research institutions in Zambia and South Africa had multiple layers of management structures to support and oversee the large number of study-employed CHWs. In South Africa, several layers of additional management structures were added over time to address challenges with staff performance (Figure 1). The initial supervision structure included the intervention manager, district managers, supervisors and CHWs. About 18 months into the intervention site managers were added, and later still, a deputy intervention manager to assist with managing staff processes whereas in Zambia the supervisory structure (intervention manager, district intervention manager, supervisors) remained consistent throughout.

Staff managers faced several challenges over the course of the intervention. For example, in Zambia, absenteeism, thought to be associated with burn out, was common. CHWs noted that delivering the door-to-door services over long distances proved to be physically taxing. Consequently, levels of absenteeism increased during the rainy season and hot summer days. In South Africa, the lack of prior work experience of many of the CHWs employed by the trial was also concerning as managers reported that it often resulted in low work ethic, leave without permission and resignations without notice (KII, South Africa, 2014). Other difficulties, experienced across both countries, included low morale, disciplinary issues, staff turnover, not reaching targets^4^ and data quality challenges. To counter these obstacles, managers had to be responsive to the daily challenges CHWs experienced.

In both countries, supervisors had weekly meetings with the CHWs to check on progress and to provide support. For quality control purposes, the supervisors conducted monthly accompanied visits to ensure that CHWs were implementing the intervention according to protocol. Supervisors also accompanied teams who were experiencing challenges related to reluctant clients, data capturing issues, and difficulties reaching study targets. In South Africa, the supervisors relayed how accompanied visits were explained to the CHWs:

The more we accompany you to the field, we are able to give you immediate feedback of how you’re doing instead of waiting until … [you] are evaluated [to] see how you’re performing. And [to only] then be told you’re underperforming (KII, South Africa, 2016).

Supervisors also conducted unaccompanied visits to participant households to verify the quality of the data recorded by CHWs. One manager explained:

I go unaccompanied in the field to assess the performance of the CHWs … The details that I get are from the clients themselves … From whatever they tell me, I will be able to assess, did [the CHWs] perform well or not (KII, Zambia, 2015)

As an additional measure, the CHWs’ supervisors reported to district-level managers, who were responsible for the overall implementation of the intervention in the respective communities. These staff accompanied CHWs in the field at least once a quarter. One district manager in South Africa said that:

[Certain] events contributed quite a lot to the improvement of the data that we’re collecting in the field… [including] accompanied evaluations that are being done, whereby the site managers and supervisors are involved, and everybody is making notes and tracking the way that CHWs perform (KII, South Africa, 2016).

An additional support structure developed from the ‘daily dashboard’, created from data CHWs uploaded to a central server on a daily basis. The managerial and data teams would then share the statistics with the CHWs. Similar to the recommendations of Assegaai *et al.* (2018), the trial used routine primary healthcare indicators to guide intervention implementation. The dashboards not only highlight challenges but also helped to visualize progress and motivate teams. Several managers also took innovative steps to support the CHW teams.

We get daily stats and, the site supervisors, they’ve created a [mobile] chat group whereby the site supervisors can see how their team is doing … every day after the shift they would send us how their teams have done. Every Monday we meet as the site managers and the district managers and then we go through the stats and then we have nice progress reports (KII, South Africa, 2016).

Managerial staff were responsive to the practical challenges CHWs experienced and took their suggestions on board. One CHW explained:

You go to household the first time around … but then they are hesitant to participate. You come and tell [supervisors] what is [happening] on the ground and that if there is intensive, intensified sensitization, like from [the community mobilisers] it will help (GD, Zambia, 2016).

Mangers also took other steps to mitigate the difficulties that CHWs encountered. In Zambia, selected CHWs were trained to be mentors to assist their colleagues with day-to-day challenges. The mentors had monthly group sessions and at least one individual session per quarter with CHWs. The CHWs also had monthly team meetings, regular training and annual team building events. In South Africa, the CHWs received psychosocial support from professional mentors who met with each of the teams on a monthly. The practical safety training was also mentioned as a supportive measure for staff in South Africa.

During interviews in 2014, many of the CHWs stated that they did not experience any support from management. However, during follow-up interviews in 2016 when many of these supportive measures were implemented, CHWs were more optimistic. One CHW in South Africa emphatically explained how their supervisors were supportive: ‘they always encourage us … I would think they support us, really!’ (GD, SA19, 2016). The Zambian CHWs also told how they received support from ‘supervisors and colleagues’ as well as from senior managers:

Support can also come from head office because there are times maybe when we are lacking on some information and we go for refresher trainings … [we receive] support from head office (GD, Z8, 2016).

Over time, CHWs in both countries reported that many of the challenges during the initial rounds of implementing the intervention were addressed, either through increased support or in collaboration with the community engagement staff who helped foster positive regard for the CHWs by the communities they served.

## Discussion

Aligned with the UK MRC process evaluation framework (Moore *et al.*, 2015), we provided detailed documentation of how the CHW-delivered components of the PopART intervention were delivered in practice. Previous papers (Floyd *et al.*, 2018; Hayes *et al.*, 2019) have shown how key target outcomes changed over the course of the trial—including increases in the proportion of PLHIV who knew their HIV status and were on ART in the interventions arms where CHWs were active. In turn, this led to higher viral suppression and lower HIV incidence in intervention arms when compared to control arms. We have focused on the activities that led to these changes and the health personnel who delivered the intervention. We have shown how CHWs were recruited, trained, managed, and supported to achieve the scale up of UTT in intervention communities.

Strengths of this analysis include that it (i) draws on multiple qualitative and quantitative data sources, (ii) from the largest trial measuring the impact of UTT on HIV incidence, (iii) in two high burden countries. Limitations to extrapolations from these findings include that these CHWs worked within a community-randomized trial and implementation dynamics may be different when delivered across a health service ‘to scale’. In addition, the Western Cape province has a generally higher resourced health service than other provinces in South Africa.

In this discussion, we reflect on how this robust description of how implementation was achieved supports our interpretation of the trial outcome findings, and how it helps provide guidance for others interested in implementing this approach in other settings.

### Fidelity of delivery

Despite challenges in rapidly delivering such an intense HIV prevention intervention, we show that it was feasible for CHWs to deliver the PopART UTT model with high fidelity during the trial. This strengthens the interpretation of the trial outcomes. We found that the cadres of workers recruited differed across the two countries, owing to divergent historical roles of CHWs and broader contextual differences. Regardless of previous experience at recruitment, CHWs across Zambia and South Africa were not familiar with novel concepts such as UTT and TasP and regular, extensive, and responsive training was needed to ensure effective delivery of the intervention. Coordinating such a large, diverse work force required intensive management structures. The role of CHWs was both physically and emotionally draining, thus keeping morale levels up added an extra task for management. Support structures were put in place to address concerns by health workers; including formalized monthly mentorship meetings and safety training in South Africa and CHW peer-peer mentorship in Zambia. Despite the trial addressing most of the challenges (i.e. better training, recognition, and structure) and ultimately leading to high retention levels amongst the CHWs, there was still high turnover amongst CHWs, particularly initially in South Africa. Low remuneration was cited as one of the main sources of job dissatisfaction among the CHWs and adjusted salaries led to higher retention.

### Key insights for transferring lessons from the PopART intervention to new settings

Similar to our findings, researchers have found that inadequate training, supervision and compensation contributed to the challenges experienced by CHWs in roles that are, by definition, physically and emotionally demanding (Di Paola and Vale, 2018; Mundeva *et al.*, 2018). In addition, CHW policies in resource constrained settings have failed to recognize the challenges associated with working in contexts of precarity that CHWs face (Swartz, 2013). CHWs are often presented in policy as the ‘cure-all’ to address shortcomings of the public health system. This positioning of CHWs means that they are often burdened with duties beyond their expertise and beyond their expectations (Colvin and Swartz, 2015). Our findings show that in addressing these concerns—providing adequate and regular training, establishing layered management structures, and ensuring consistent emotional and moral support—CHWs were better adapted and prepared to conduct the demanding work associated with community-based care.

We provide the following recommendations on how to best deploy CHWs in the effort to implement UTT in sub-Sahara African or low- and middle-income countries: health systems should (i) tap into local networks and knowledge systems of experienced CHWs; (ii) provide regular, responsive and context-specific training on HIV treatment guidelines, including the expanded use of HIV treatment as prevention; (iii) provide sufficient staff supervision, including regular meetings; accompanied and unaccompanied field visits; ensuring support structures are in place; and (iv) ensure remuneration for CHWs is competitive and staff safety and emotional wellbeing are assured. Importantly, we also encourage policymakers to acknowledge the diversity of CHWs and to be responsive in terms of the employment, training, supervision, and emotional support of front-line workers.

In both Zambia and South Africa, a network of CHWs exist that are able to provide services beyond HIV testing, care, and referrals. Through integrating HIV services into wider health screenings and services, health systems would be able to capitalize and build on existing skills in the roll-out of UTT.

In both countries, current CHW systems would benefit from implementing the recommendations noted above. Specifically, as HIV services are expanded to include, for instance, HIV self-testing, adequate training responsive to community concerns will be needed to ensure efficient service roll-out (Bwalya *et al.*, 2020). Health systems in Zambia and South Africa experience challenges in linking patients to facilities. Through incorporating expanded, localized training, providing supportive staff supervision, CHWs will be better able to address and support the needs of community members and health systems. In South Africa, CHWs received substantially more money and worked longer shifts than existing CHWs. This is in line with planned shifts for the expansion of CHW-driven service delivery and offers a model for the required training, oversight and expanded career pathways for this cadre.

The HIV test and treat initiative recently adopted in Zambia and South Africa will place an increased demand on the already strained human resources in healthcare settings in both countries. Further data are required on how best to optimize cadres of CHWs to mitigate this strain. Implementing community- and home-based HIV service delivery requires expanding the scope of CHWs to include direct service delivery, managing implementation processes for the programme, and collecting monitoring information to ensure service quality. In parallel, CHW programme investment requires commensurate resource investment. We suggest that urgent priority be given to systematic evaluations of mechanisms to support maximum economic efficiency and quality in the services offered by CHWs such that national programmes can cost and include these into the wider service delivery budgets.

## Footnotes

1 The enumeration database was created in partnership with health providers to collect demographic data for all health workers. All health workers on the enumeration lists were invited to participate in the Stigma Survey.

2 Study communities are anonymized by numbers with Z1–Z12 in Zambia, and SA13–SA21 in South Africa.

3 Trial implementers attempted to pair men and women CHWs to counter these and other challenges.

4 CHW teams were initially required to provide HIV testing and care services at eight households per day (minimum). This was lowered as the focus shifted to quality over quantity, although managers would check in with teams visiting fewer than five households on a given day.
