# Corrigendum to: community-based health workers implementing universal access to HIV testing and treatment: lessons from South Africa and Zambia—HPTN 071 (PopART)

**DOI:** 10.1093/heapol/czab070

**Published:** 2021-06-29

**Authors:** Lario Viljoen, Tila Mainga, Rozanne Casper, Constance Mubekapi-Musadaidzwa, Dillon T Wademan, Virginia A Bond, Triantafyllos Pliakas, Chiti Bwalya, Anne Stangl, Mwelwa Phiri, Blia Yang, Kwame Shanaube, Peter Bock, Sarah Fidler, Richard Hayes, Helen Ayles, James R Hargreaves, Graeme Hoddinott

In the originally published version of this manuscript, the spelling of the co-author’s name was incorrect. The name should be “Mubekapi-Musadaidzwa” but is listed as “Mubekapi-Mu**za**daidzwa”. This error has now been corrected online.

